# An evaluation of the interaction of place and community-based participatory research as a research methodology in the implementation of a sexually transmitted infection intervention for Greenlandic youth

**DOI:** 10.3402/ijch.v75.32239

**Published:** 2016-12-09

**Authors:** Elizabeth Rink

**Affiliations:** Department of Health and Human Development, Montana State University, Bozeman, MT, USA

**Keywords:** sexually transmitted infections, community-based participatory research, place, research methods

## Abstract

Newly emerging research suggests that the actual physical location of a study and the geographic context in which a study is implemented influences the types of research methods most appropriate to use in a study as well as the study's research outcomes. This article presents a reflection on the extent to which place influenced the use of community-based participatory research (CBPR) as a research methodology in the implementation of an intervention to address sexually transmitted infections in Greenland. An evaluation of the interaction between place and CBPR suggests that the physicality of place influenced the intervention's successes and challenges. Future research that uses CBPR as a research methodology in sexual and reproductive health research in the Arctic warrants situating the research design, implementation and outcomes within the context of place.

In health sciences research, much attention has been given to understanding the extent to which a range of individual, family, social, cultural and environmental factors interact within a place to produce a research outcome (1–11). However, the “where” of the research and how the actual place of a research study influences the research methodology has received less attention (12–14).

The purpose of this article is to present a reflection on how place influenced a 3-year community-based participatory research (CBPR) educational intervention study addressing sexually transmitted infections (STIs) in Greenland. The study, called *Inuulluataarneq* (Having the Good Life), involved 15–18 year-old Inuit youth and their parents/guardians and took place in three Greenlandic communities. In this article, place is defined as a distinct physical location in which individuals’ and families’ behaviours are associated with a collection of particular meanings, beliefs, practices, values and feelings (13). The use of reflection is important to the research field as it allows the researcher to do more than report on the research findings but to also assess, process, explain and question how the research outcomes were ultimately constructed (15). Specifically, reflectivity is used in this context to explore the extent to which place made a difference in *Inuulluataarneq*'s use of CBPR as a research methodology.

## *Inuulluataarneq* (Having the Good Life): the communities

**Fig. 1:**
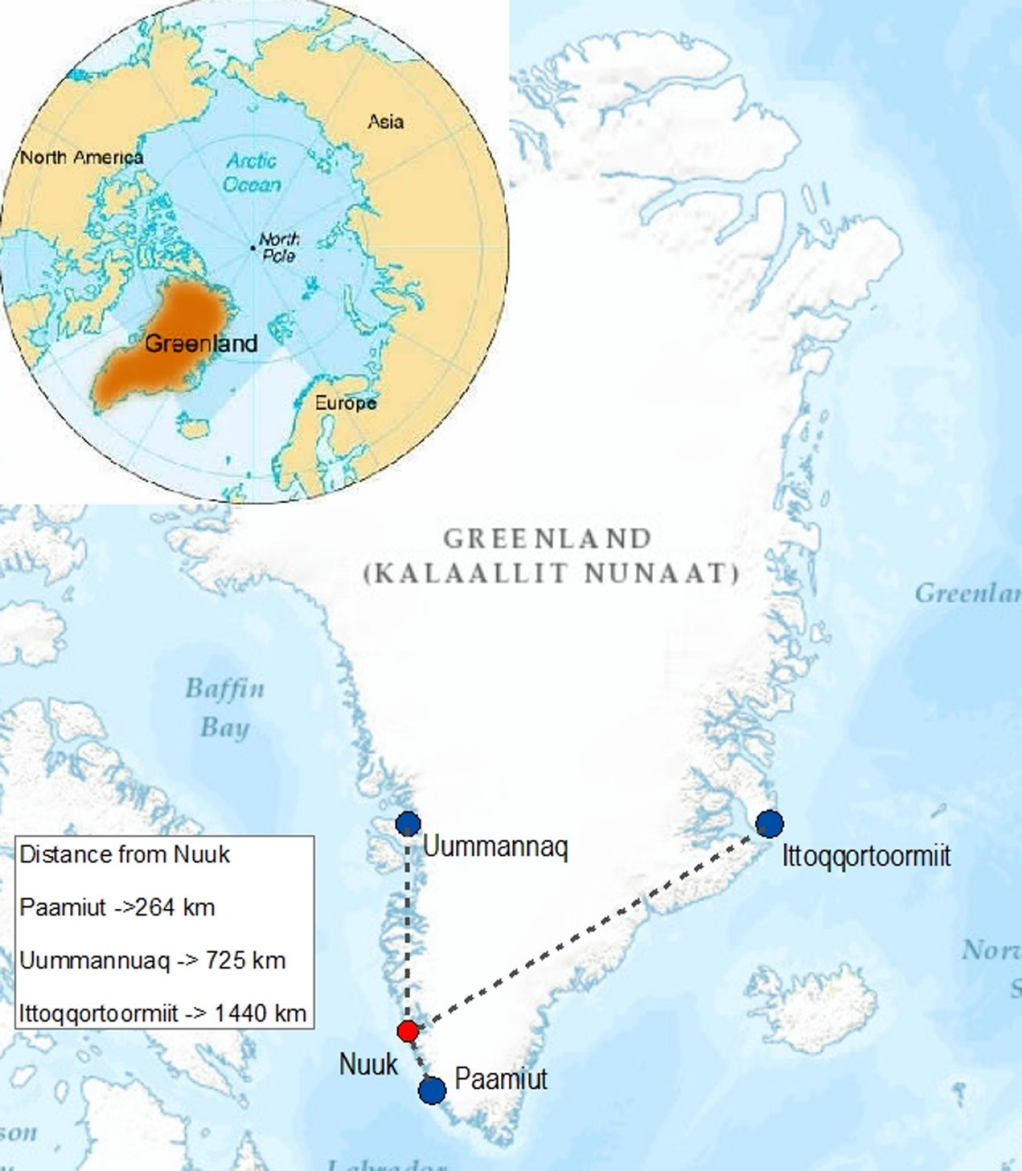
Map of Inuulluataarneq Research Sites in Relationship to Nuuk. *Source:* ArcMap 10.3.1., Esri, HERE, DeLorme, Intermap, increment P Corp, GEBCO, USGS, FAO, NPS, NRCAN, GeoBase, IGN, Kadaster NL, Ordnance Survey, Esri Japan, METI, Esri China (Hong Kong) swisstops, MapmyIndia, Openstreetmap contributors, and the GIS User Community.

Three communities participated in *Inuulluataarneq*. Paamiut (population=1,619) is located in the southwestern part of Greenland below the Arctic Circle. Uummannaq (population=1,299) is located in the northwest of Greenland above the Arctic Circle (16). Ittoqqortoormiit (population=469) is located in the northeast of Greenland also above the Arctic Circle. The University of Greenland located in Nuuk was the central organizing institution for the project (Fig. 1) (17).

*Inuulluataarneq*'s research sites had several similarities. Paamiut, Uummannaq and Ittoqqortoormiit had traditional Greenlandic kinship networks with large nuclear and extended families that relied on each other for economic and social support (18–21). About 5% of the population in Paamiut, Uummannaq and Ittoqqortoormiit were Danish with the majority of the population being of Inuit descent. Greenlandic was the primary language spoken in the households. In all three of *Inuulluataarneq*'s research sites, Lutheranism was the predominate religion (22,23).

Differences in the three communities were found in the communities’ histories and economies. For example, the area that is now Paamiut has had inhabitants of Inuit descent since 1500 BC. Paamiut was established as a town in 1742, shortly after Greenland was colonized by Denmark, and was *Inuulluataarneq*'s study site located the closest to Nuuk (264 km). In Paamiut, people make their living primarily on hunting and fishing. Paamiut is home to a large fish factory and had prosperous economic history with a booming cod fishing and processing industry in the later part of the twentieth century. At the time *Inuulluataarneq* was implemented in Paamiut, the unemployment rate was 13.1%. Uummannaq was established in the mid-1700s by local hunters from the neighbouring Ikerasak area because of the prosperous whale hunting in the area that attracted whalers from around the world. Like Paamiut the main industry in Uummannaq is hunting and fishing. The unemployment rate in Uummannaq was 6.4% at the time of the study. Uummannaq is 724 km north of Nuuk and the research site that was the second farthest away from Nuuk. Ittoqqortoormiit was established in 1925 primarily by the Danish with a small number of Inuit families from Tasiilaq (also on the east coast of Greenland). Although people in Ittoqqortoormiit hunt and fish, the economy in Ittoqqortoormiit is based on the public administration and social services infrastructure. During the study, there was no fish factory or large industry in Ittoqqortoormiit, and the unemployment rate was 19.4%. Ittoqqortoormiit is 1,440 km northeast of Nuuk and was the research site farthest away from Nuuk (17,24).

## The influence of place on CBPR as a research methodology

*Inuulluataarneq* used a CBPR framework as well as CBPR guidelines established specifically for working with Indigenous populations (16,25–27). CBPR is an iterative, flexible, inclusionary process in which communities and researchers work side by side as equal partners to address a health disparity of relevance to both the community and the researchers. Increasingly, CBPR is seen as a research framework for indigenizing research methodologies in order to promote relevant research studies and increase positive health outcomes for Indigenous populations because of CBPR's emphasis on inclusion, respect, reciprocity and circular process, all of which are principles inherit in how Indigenous peoples view their world (28–31). CBPR is also viewed as a preferred research framework for Indigenous populations because often these communities are physically isolated, small in size and culturally unique (32,33). Given this understanding of CBPR, it seemed a well-suited research methodology for implementing *Inuulluataarneq*.

In the case of *Inuulluataarneq*, researchers from outside Greenland were invited to the three communities to work on a project related to STIs. Community advisory boards were established in each community. Community outreach workers from each of the communities were hired from within the community to work with the community advisory boards and researchers. Research team members spent substantial amounts of time (several weeks to a couple of months) in each community working with the community outreach workers, community advisory boards and other key stakeholders in each community to develop, implement and evaluate *Inuulluataarneq*. Results from *Inuulluataarneq* showed a relationship between decreased rates of chlamydia and increased communication between parents and guardians about topics related to sex (13).

Despite *Inuulluataarneq*'s adherence to CBPR principles and practices, as well as repeated discussions with community members about what CBPR was and how a CBPR framework is implemented, at every stage of *Inuulluataarneq*'s roll-out, including creating the study's intervention components, implementing the intervention, collecting the data, evaluating the data and deciding on the best ways to share the study results with others, *Inuulluataarneq*'s CBPR methodology was experienced differently in each research site.

An evaluation of why *Inuulluataarneq*'s CBPR methodology was experienced differently in Paamiut, Uummannaq and Ittoqqortoormiit suggests a connection to the “where of method.” Paamiut, the closest community to Nuuk (264 km), implemented *Inuulluataarneq*'s CBPR framework most effectively. The proximity of Paamiut to Nuuk allowed for frequent communication and reliable travel to and from the study site by the researchers. The community outreach worker from Paamiut was also able to travel reliably and within a couple of hours to Nuuk in order to work with members of the research team at the University of Greenland. This consistent contact enabled the community members in Paamiut and the researchers to establish professional as well as personal relationships with each other. As stated earlier, these types of trusting relationships, which were made possible by the closeness and easy access of the researchers to the community, are a mainstay of CBPR and foundational to the success of any CBPR project.

In addition, at the time *Inuulluataarneq* was implemented in Paamiut, the community was engaged in a longitudinal community-wide research project with Danish and Greenlandic academics called *Paamiut Asasara*. Because of this established involvement with research as well as a familiar pattern of outsider movements coming and going from Paamiut, community members understood that researchers come and go from a study site. This community familiarity with research made establishing relationships to maximize *Inuulluataarneq*'s CBPR framework relatively efficient and smooth.

Uummannaq, the second farthest research site away from Nuuk (725 km), also successfully implemented *Inuulluataarneq*'s CBPR framework. Similar to Paamiut, community members in Uummannaq were familiar with outsiders and their movements of coming and going from the town. However, travel to Uummannaq was more time-consuming and unpredictable than Paamiut. In order to adhere to the tenets of CBPR and find balance between the community's understanding of working with outsiders and the issues of distance and travel, the researchers involved in *Inuulluataarneq* stayed in Uummannaq for longer periods of time than in Paamiut. The researchers’ ability to prolong their presence in Uummannaq and adjust *Inuulluataarneq*'s implementation facilitated the relationships necessary to create a continuous dialogue with the community members about *Inuulluataarneq*.

Ittoqqortoormiit, the farthest away from Nuuk (1,440 km) was the most difficult community for implementing *Inuulluataarneq*'s CBPR framework. The logistical challenges of getting to and from Ittoqqortoormiit, including the travel time and the unpredictability of the weather, were great. For example, during the implementation of *Inuulluataarneq*, helicopter flights to Ittoqqortoormiit were once a week. In poor weather, a weekly flight may be cancelled, and in one instance, members of the research team were not able to leave Ittoqqortoormiit for an extra 2 weeks because of weather delays. These logistical challenges highlighted the isolated nature of the community. The remoteness of Ittoqqortoormiit seemed to have a negative impact on relationship development and maintenance between the researchers and community members in that people in Ittoqqortoormiit were less familiar with outsiders coming and going. If outsiders did come, community members were reluctant to get involved with them because they were not familiar or comfortable with a pattern of outsiders coming and going from the community. This awkward dynamic between *Inuulluataarneq*'s research team and the community members made it difficult to establish the trusting relationships that are central to CBPR. This made the implementation of *Inuulluataarneq* in Ittoqqortoormiit ineffective.

## An examination of the interaction between place and CBPR

A possible explanation for the contrasting experiences with CBPR observed in *Inuulluataarneq*'s study sites is the concept of the proximity paradox (34). The proximity paradox refers to the contradiction that arises between using CBPR with Indigenous populations in remote or isolated geographic locations and the difficulty of conducting CBPR projects specifically because of the geographic remoteness and isolation. CBPR is built on the foundational premise of trust and the importance of community members and researchers building and maintaining trusting relationships with each other (35). The establishment of trusting community–researcher partnerships takes time, meaning not only length of time but also time together which in geographically difficult to access communities is more of a challenge than in communities that are logistically easy to access. Thus, the proximity paradox emerges.

Previous research assessing the proximity paradox concluded that there are contradictions in using CBPR as a method to conduct research with Indigenous populations in remote locations (34). Namely there is a need to use CBPR with hard to reach, culturally distinctive Indigenous populations. However, trusting relationships are difficult to establish and maintain with these populations because of the physical distance between the communities and the researchers. The potential inability to create trusting relationships jeopardizes the effectiveness of CBPR and thereby risks the effectiveness of the research outcomes. In contrast, Indigenous populations that are closer and easier for researchers to access may not have as many unmet needs as more distant Indigenous communities, but there is a more fluid potential to establish trusting relationships. This fluidity of relationships increases the effectiveness of a study's implementation and outcomes.

On examination of the interaction of the physical location of *Inuulluataarneq*'s research sites with CBPR as a research methodology, the proximity paradox provides insights into the *Inuulluataarneq*'s successes and challenges. To summarize, the close proximity of Paamiut to the University of Greenland in Nuuk created a successful situation for community members in Paamiut and members of *Inuulluataarneq*'s research team to establish trusting and productive relationships, which in turn established the success of *Inuulluataarneq* in Paamiut. The success of *Inuulluataarneq* in Paamiut was also due to the community already having experiences conducting research and working with researchers as well as the community's comfort level with researchers coming and going from Paamiut. In Uummannaq, which was not close to the University of Greenland, *Inuulluataarneq* was also successful. The success of *Inuulluataarneq* in Uummannaq was related to the researchers spending longer periods of time in the community in order to compensate for the distance away from Nuuk to build and maintain relationships with the community members. Uummannaq also had a history of outsiders coming and going from their community, which contributed to the communities’ comfort level with the travel pattern of the research team members. Thus, we found that in Paamiut and Uummannaq, the ability of the research team members to establish trusting relationships and work closely with community members to design and implement *Inuulluataarneq* resulted in the community's ability to recruit research participants, collect relevant data, interpret the data in a way that was useful for them, and to share the study's results with a broader audience in a manner that they believed appropriate and reflective of how to address STIs in Greenland.

In contrast, Ittoqqortoormiit was the least proximal to the University of Greenland, which made creating the time to travel and stay in Ittoqqortoormiit to implement *Inuulluataarneq*'s CBPR framework particularly arduous. Furthermore, the community of Ittoqqortoormiit had a history of suspiciousness towards outsiders coming and going from their community. The combination of these two factors was key to *Inuulluataarneq*'s research team not being able to implement the study in Ittoqqortoormiit regardless of their attempts to adjust travel and fieldwork plans to work with community members on the study.

## Conclusions

*Inuulluataarneq* offers a perspective on the extent to which the geographic location of study sites situated in the Arctic can impact research methods and, subsequently, research outcomes. *Inuulluataarneq*'s success at increasing youth–parent/guardian sexual health communication and reducing chlamydia were linked to the study's CBPR orientation (36–39). Proponents of CBPR attest to its methodological strengths, including face-to-face interactions and a community of focused philosophy, with small, isolated communities, such as with Indigenous Arctic populations, in order to increase community awareness of research and community members’ participation in research studies and enhance the interpretation and understanding of the research findings (40–44). However, the use of CBPR with remote Indigenous Arctic communities is not just about relationships and process, but how these relationships and processes play out within the context of place and impact the ability to conduct research.

Although it can be argued that relationships are inaugural to any CBPR project regardless of the topic being researched, sexual and reproductive health research is highly personal and potentially emotional because of the intimate nature of the data collected (39). Furthermore, sexual and reproductive health research, particularly research that addresses intimate partner violence and sexual abuse, in small, isolated Arctic populations may be volatile because of the close knit interpersonal and familial relationships within the communities. The use of CBPR with projects on sensitive issues, such as those confronted in sexual and reproductive health, facilitates the establishing of trust between the researched and the researcher as well as developing an understanding of the context in which the sexual and reproductive health research is taking place. Overall, *Inuulluataarneq* seemed to be successful in creating a dialogue within communities through the use of CBPR about topics related to sexual and reproductive health. The interaction that this dialogue generated seemed to be effective in raising awareness of the social, emotional and behavioural factors contributing to STIs among youth (36,38).

Understanding how place shapes research methodology can contribute to fruitful research outcomes and, ultimately, healthier people in the Arctic. As demonstrated in *Inuulluataarneq* the “where of method” influenced the research team's ability to develop relationships with community methods, which in turn influenced the effectiveness of CBPR as a research methodology. Subsequently the effectiveness of CBPR influenced *Inuulluataarneq*'s research outcomes.

More attention could be given to understanding the interaction of CBPR as a research methodology and the actual place where a CBPR study is implemented. Researchers may consider using community readiness models in addition to CBPR principles and practices to determine how prepared a community is to implement a CBPR study (45–47). As experienced in *Inuulluataarneq*, simply because the communities invited the researchers to their communities to study STIs did not in turn mean that they were actually prepared and ready to implement a CBPR study. In addition, researchers working in isolated Arctic communities using CBPR may consider working in fewer communities in order to focus their resources and gain a deeper perspective of the complexity of issues facing a community as it relates to a particular health topic. For example, *Inuulluataarneq* may have had a different outcome in Ittoqqortoormiit if the research team could have concentrated its efforts there as opposed to spreading their work over three different communities in three different parts of Greenland. The experience of *Inuulluataarneq* in Ittoqqortoormiit highlights the importance of researchers and community members taking into consideration the “where” of place and its impact on the research process when the study site is not close to central, more easily accessible Arctic hubs. In conclusion, there is a growing body of research suggesting alternative research strategies for working with small populations as those found among Indigenous groups in the Arctic. This current research moves away from the emphasis on quantitative data analysis and use of sample size and statistically significance, and, instead, focuses on the use of narratives and storytelling to understand Indigenous world views (32,33,48,49). Given that populations in the Arctic are either declining or remaining stable with no growth rate, understanding the complexity of how place interacts with sexual and reproductive health research methods and outcomes is warranted.
